# Comparison of Experimental Strategies to Study l-Type Amino Acid Transporter 1 (LAT1) Utilization by Ligands

**DOI:** 10.3390/molecules27010037

**Published:** 2021-12-22

**Authors:** Johanna Huttunen, Mahmoud Agami, Janne Tampio, Ahmed B. Montaser, Kristiina M. Huttunen

**Affiliations:** School of Pharmacy, Faculty of Health Sciences, University of Eastern Finland, P.O. Box 1627, FI-70211 Kuopio, Finland; johanna.huttunen@uef.fi

**Keywords:** l-type amino acid transporter 1 (LAT1), targeted drug delivery, transporter study methodology, intracellular kinetics, amino acid homeostasis, transporter regulation

## Abstract

l-Type amino acid transporter 1 (LAT1), expressed abundantly in the brain and placenta and overexpressed in several cancer cell types, has gained a lot of interest in drug research and development, as it can be utilized for brain-targeted drug delivery, as well as inhibiting the essential amino acid supply to cancer cells. The structure of LAT1 is today very well-known and the interactions of ligands at the binding site of LAT1 can be modeled and explained. However, less is known of LAT1′s life cycle within the cells. Moreover, the functionality of LAT1 can be measured by several different methods, which may vary between the laboratories and make the comparison of the results challenging. In the present study, the usefulness of indirect *cis*-inhibition methods and direct cellular uptake methods and their variations to interpret the interactions of LAT1-ligands were evaluated. Moreover, this study also highlights the importance of understanding the intracellular kinetics of LAT1-ligands, and how they can affect the regular function of LAT1 in critical tissues, such as the brain. Hence, it is discussed herein how the selected methodology influences the outcome and created knowledge of LAT1-utilizing compounds.

## 1. Introduction

l-Type amino acid transporter 1 (LAT1), commonly referred to as the “large neutral amino acid transporter 1”, less commonly referred to as the “L-leucine preferring amino acid transporter 1”, is a pH and sodium-independent antiporter. This heterodimeric protein complex is an essential carrier of large, neutral, aromatic, or branched L-amino acids (e.g., L-Leu, L-Phe, L-Tyr, L-Trp, L-His, L-Met, L-Ile, and L-Val) and some amino acid mimicking drugs (e.g., L-dopa, gabapentin, baclofen, and melphalan) [1,2,3,4]. Curiously, it has been noted that LAT1 is stereospecific, preferring L-forms over the D-forms, and having a higher affinity for its substrate amino acids than the other transporters in the periphery belonging to the same LAT-family [5]. The transport rate of LAT1 substrates has been postulated to be controlled by the concentration of intracellular substrates, LAT1 having a higher affinity for intracellular amino acids than amino acids in the extracellular space [6,7]. Nevertheless, LAT1 is a high affinity–low-capacity transporter, i.e., the K_m_ and V_max_ for LAT1 substrates are typically very low (K_m_ of 13–28 µM and V_max_ of 2–6 pmol/10^6^ cells/min for human isoform) [3,8].

LAT1 consists of a light chain (*SLC7A5*) and a CD98 heavy chain (4F2hc; *SLC3A2*), which are linked together via a disulfide bond. The exact role of CD98 is not fully understood, but it has been proposed that it chaperones the complex’s localization to the cell membrane and affects the LAT1′s transportation activity [4,9,10]. Before the structure of LAT1 was solved by cryo-electron microscopy (Cryo-EM) [9,11], several different homology models, structure-activity relationships (SAR), protein-ligand models, and pharmacophore models were proposed [5,12,13,14,15,16,17]. However, although the transport mechanism of LAT1 at an atomistic level has been shown with molecular dynamics (MD) simulations [18], and it is nowadays well accepted that LAT1 has two major states—inward-open and outward-open phases [5]—further research is required to explain the rearrangement between these states and how structural differences of LAT1-ligands can affect the transport process through the LAT1 cavity.

LAT1 is distributed throughout the body, and it is highly expressed in tissues that require a high amino acid supply, such as the brain and placenta, but it is also found to a lesser extent in other tissues [2,3,19,20]. Moreover, LAT1 is needed to meet the increased demand for amino acids in proliferating cancer cells; therefore, it is upregulated in various types of tumors [8,21,22]. Similarly, certain immune cells can upregulate LAT1 expression on their cell surface upon their activation [23,24]. The protein complex is found mainly in the basolateral membrane of polarized epithelia, with the exception of the blood–brain barrier (BBB), where it is localized on both luminal and abluminal sides. It has also been found that ubiquitylation of LAT1′s *N*-terminal tail induces its endocytosis and degradation [25]. Moreover, it has been demonstrated that the lysosomal protein, LAPTM4b, can recruit LAT1 to lysosomes [26]. This function has been proposed to be associated with increased L-Leu uptake into the lysosomes and subsequent activation of mammalian target of rapamycin 1 (mTORC1), a regulator of cell growth. Interestingly, some upstream regulators have also been recognized in distinct cancer cells [27] and silencing these activators have been shown to decrease L-Leu uptake and subsequent mTOR activity and proliferation of cancer cells. However, very little is known about how LAT1 can be upregulated in healthy cells.

In the present study, the aim was to evaluate the usefulness of different methods and their variations to study LAT1 utilization by its ligands with LAT1-expressing immortalized mouse microglia (BV2) [20]. We will compare and discuss what type of information can be concluded from the most commonly used methods, including *cis*-inhibition studies (binding of a ligand to LAT1 is estimated by its efficiency to inhibit radiolabelled LAT1-probe substrate) as well as direct cellular uptake studies of compounds being measured from the cell lysates. We will also give insights into how more profound knowledge can be attained if one is relying only on one particular method. Lastly, we encourage other types of studies, not only LAT1-utilization, to fully understand how LAT1-utilizing compounds behave intracellularly and how they affect the cellular functions, including the function of LAT1 itself.

## 2. Results

### 2.1. Selection of Studied Compounds

**Figure 1:**
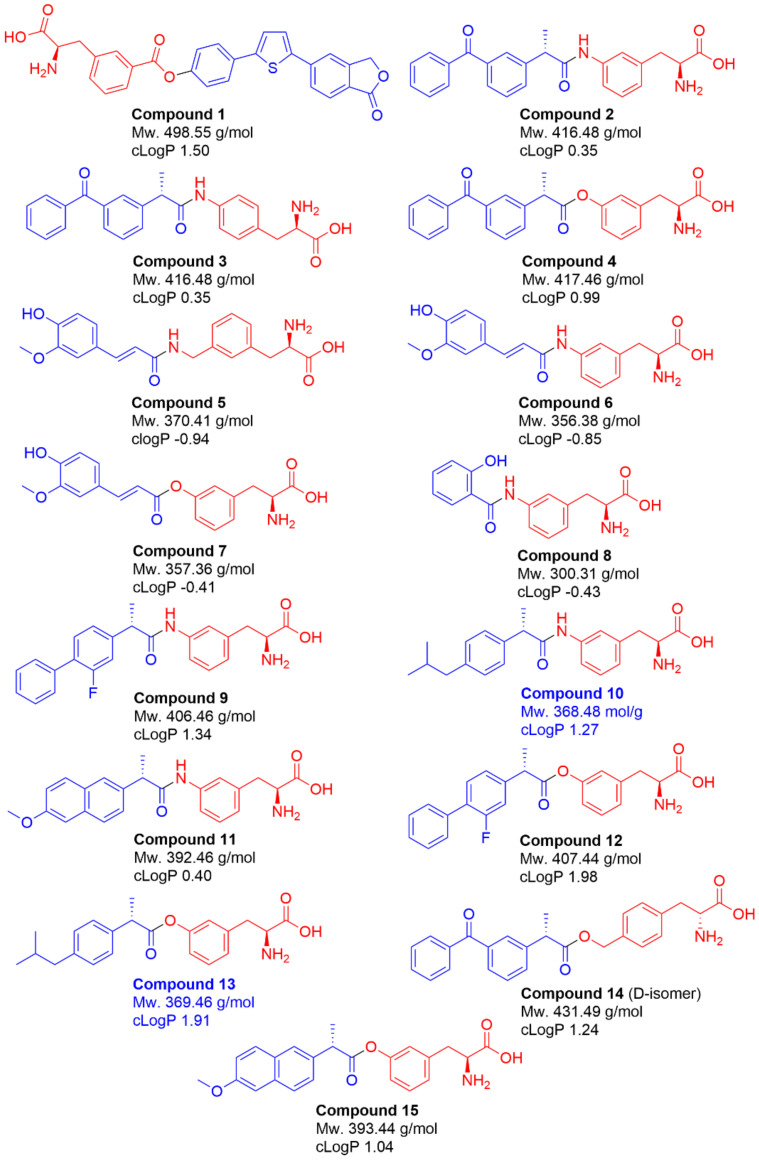
Chemical structures and properties of studied LAT1-utilizing compounds **1**–**15**. The parent drugs are indicated with a blue color and the promoieties with a red color.

LAT1-utilizing prodrugs **1**–**15** (Figure 1) were selected for this study based on their ability to utilize LAT1 expressed in cells used in our previous studies. Therefore, the syntheses of these compounds are not described herein as they have been reported earlier [28,29,30,31,32,33]. These compounds include prodrugs of an investigational immunosuppressive agent, perforin inhibitor (**1**), non-steroidal anti-inflammatory drugs (NSAIDs), ketoprofen (**2**–**4**, **14**), salicylic acid (**8**), flurbiprofen (**9** and **12**), ibuprofen (**10** and **13**) and naproxen (**11** and **15**), as well as the polyphenolic antioxidant, ferulic acid (**5**–**7**). Notably, all prodrugs were pure L-amino acid stereoisomers, except compound **14**, which was a pure D-amino acid stereoisomer instead (Figure 1). In addition, all NSAID prodrugs (compounds **2**–**4**, **9**–**15**) were prepared from their pure *S*-enantiomers. Prodrugs of ferulic acid (compounds **5**–**7**) were mixtures of *E*- and *Z*-enantiomers, which were interconvertible over time. The molecular weights of the studied compounds varied from 300.31 g/mol to 498.55 g/mol and their calculated logP values were within the range of −0.41–1.98 (Figure 1). All the LAT1-utilizing prodrugs that have the amide prodrug bond (compounds **2**, **3**, **5**, **6**, **8**–**11**) are highly stable in biological media in vitro, but not in vivo, as well as in 0.1 M NaOH (used for the cell lysing) while the prodrugs with ester bond (compounds **1**, **4**, **7**, **12**–**15**) are bioconverted to their parent drugs with varying rates both in biological media as well as in 0.1 M NaOH [28,29,30,31,32,33]. Therefore, both prodrug and the released parent drug proportions were quantified and taken into account when calculating concentrations transported into the cells.

### 2.2. LAT1-Utilizing Compounds Can Inhibit Cellular Amino Acid Uptake

LAT1-utilization, and particularly binding of ligands to LAT1, can be studied by incubating the studied ligands with radiolabelled LAT1-probe substrates, e.g., [^14^C]-L-Leu, [^14^C]-L-Phe, [^3^H]-L-His, [^3^H]-L-Trp, and [^3^H]-L-gabapentin. As there is a variety of radiolabelled probe substrates that can be used, it raises the question of whether these *cis*-inhibition studies are comparable between the laboratories. Therefore, in the present study, we wanted to evaluate if there is a significant difference in the inhibition among the used radiolabelled probe substrates in these *cis*-inhibition studies. Thus, the ability of the selected LAT1-utilizing compounds (**1**–**15**) to inhibit [^14^C]-L-Leu, [^3^H]-L-Met, [^3^H]-L-Trp, and [^3^H]-L-kynurenine (Kynu) was studied in immortalized mouse microglia (BV2), which are known to highly express LAT1 [20]. The probe substrates were selected to contain small and flexible amino acids, such as L-Leu and L-Met, but also bigger and more rigid compounds, such as L-Trp and amino acid-mimetic L-Kynu. According to the results that are presented in Table 1, no major differences stemmed out from the inhibition of different LAT1-probe substrates. Curiously, most of the studied compounds had higher IC_50_ values of L-Trp uptake than other probe substrates; However, the difference was not major and the inhibition order by the compounds **1**–**15** was more or less the same with all radio-probes. Therefore, even though the results between the laboratories using different radio-probes cannot be exclusively compared, some rough conclusions, such as those discussed below, can be made.

LAT1- ligands that are relatively large and/or rigid compounds (compounds **1**, **2**, **9**, **10**, **11**, and **12**; Figure 1), inhibited the uptake of probe substrates most effectively and, thus, can be considered to have a good LAT1-binding affinity (IC_50_ < 16 µM). Notably, unstable ester derivatives (compounds **4**, **7**, **13**, **14**, and **15**) were found to inhibit the uptake of LAT1-probe substrates with variable IC_50_ values (ranging from 3 µM to over 100 µM), most likely due to the premature bioconversion on the cell surface on cells. Similarly, the smallest and most flexible derivatives, compounds **5**, **6**, and **8** (Figure 1), were not able to inhibit the LAT1-probe substrate uptake very effectively. The smallest LAT1-utilizing compounds were also less lipophilic, having negative cLogP values (Figure 1). However, no direct conclusion of the relation between lipophilicity and LAT1 affinity can be drawn. Lastly, compounds having unfavorable structures, such as attaching the parent drug (ketoprofen) to the *para*-position of the aromatic ring of amino acid residue (compound **3**) resulted in lower affinity and higher IC_50_ values, as expected according to the literature [16,17,34,35].

Next, it was evaluated whether *cis*-inhibition studies can be used to determine whether LAT1-ligands are substrates or inhibitors (binders, and not transported into the cell via LAT1). For this purpose, the inhibition type of [^14^C]-L-Leu was studied by incubating 0.010–0.20 µM [^14^C]-L-Leu uptake in the presence of LAT1-utilizing compounds (**1**–**15**) at the concentration corresponding to their IC_50_ values (3–53 µM; Table 2). All the studied LAT1-utilizing compounds decreased V_max_ of [^14^C]-L-Leu uptake; however, curiously, they either increased or decreased affinity (K_m_) of [^14^C]-L-Leu for LAT1 (Table 2). Thus, it can be concluded that bigger substrates, such as the studied compounds, behaved more like non-competitive or mixed type inhibitors to [^14^C]-L-Leu uptake. Therefore, utilizing only radio-labelled probe substrates, neither the LAT1-mediated transport process into the cells nor the differentiation of LAT1-substrates from inhibitors cannot be concluded. Thus, more sophisticated methods, such as cellular uptake of compounds analyzed directly from the cell lysates by LC-MS methods, are needed to be used.

### 2.3. Cellular Uptake of Compounds via LAT1 Is Time- and Concentration-Dependent

**Figure 2:**
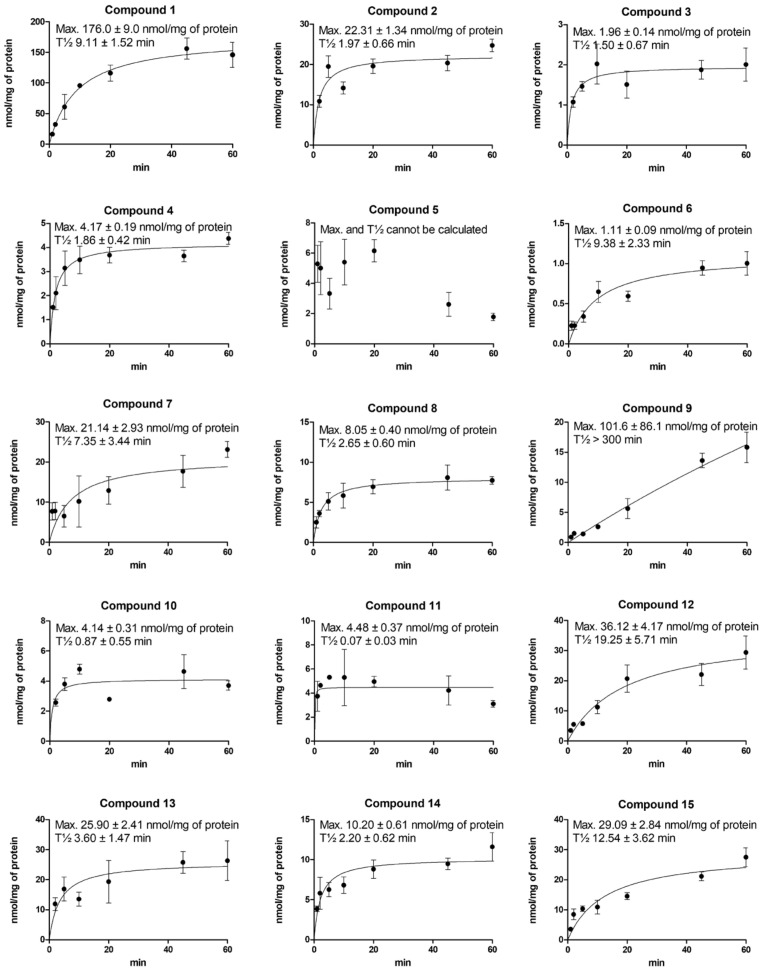
The cellular uptake (nmol/mg protein) of 100 µM LAT1-utilizing compounds into immortalized mouse microglia (BV2) plotted against the uptake time (1–60 min). The data are presented as mean ± SD, *n* = 3.

To confirm that the studied LAT1-utilizing compounds (**1**–**15**) are substrates and not only binders of LAT1, cellular uptake into immortalized mouse microglia (BV2) was studied in a time-dependent manner; 100 µM compounds were incubated 1–60 min and their concentrations (or released parent drugs) were analyzed from the cell lysates by LC-MS/MS methods. According to Figure 2, compounds **5**, **10**, and **11** were revealed to be LAT1-binders rather than substrates, as their uptake was not increased with time (T½ < 1 min). Compounds **5**, **10**, and **11** were most likely either metabolized or transported out of the cells as their uptake was decreased over the studied period of time. All the other compounds proved, in turn, to be LAT1-substrates and transported effectively inside the cells. Compound **5** was the only studied compound that had a methylene linker between the prodrug carbonyl carbon and the aromatic ring of the amino acid residue. This pushes the carbonyl carbon further away from the amino acid residue, which may be a critical distance for the transformation from the outward open state to the inward open state, making the compound more like a binder rather than a substrate. The parent drug moiety is also relatively small with this compound (total Mw. 370.41 g/mol), similar to the other binders, compounds **10** and **11** (368.48 and 392.46 g/mol, respectively). On the other hand, the clogP values of these compounds varied as much as with the substrates **1**–**4**, **6**–**9**, and **12**–**15** (from −0.94 to 1.27). Moreover, the size itself cannot explain the differences among substrates and binders, since most of the known clinically used drugs, such as gabapentin and L-dopa, and all the LAT1-utilizing amino acids are relatively small. Therefore, it is important to understand the structural relationships regarding the conformational changes of the protein during the transport process. For that purpose, computational methods are needed to be combined with experimental data, e.g., by using molecular dynamics simulation assays [36].

To understand the cellular uptake of studied LAT1-utilizing compounds (**1**–**15**) in more detail, a concentration-dependent uptake was performed with immortalized mouse microglia (BV2) by incubating the compounds over a range of 1–200 µM for 30 min and analyzing the amounts by LC-MS/MS methods. With this method and only after the subsequent Eadie–Hofstee analysis, it was clearly seen that all the studied compounds were able to utilize not only LAT1, but also another transport mechanism (Table 3, Figure S1). We have previously reported that organic anion transporting polypeptides (OATP) can carry LAT1-utilizing compounds at higher concentrations or when LAT1 is occupied into the human breast cancer cells (MCF-7) [37]. However, we have concluded earlier that, since OATPs are low-affinity–high-capacity transporters, their possible physiological role as carriers of LAT1-utilizing compounds is minor. According to the intrinsic clearance (CL_int_ = V_max_/K_m_) calculated for both transport mechanisms of each compound in this study, the same can also be concluded in the present study with immortalized mouse microglia (BV2) (Table 3); all the CL_int_ values for LAT1-mediated transport were much higher compared to the CL_int_ values of the secondary transport mechanism.

The LAT1-utilization for the cellular uptake of the studied compounds **1**–**15** was demonstrated by utilizing LAT1-selective inhibitor (KMH-233) [38]. The uptake of compounds **1**, **2**, **3**, **4**, **6**, **7**, **8**, **9**, **12**, **13**, and **15** was inhibited significantly in the presence of LAT1-inhibitor in BV2 cells, while with compounds **5**, **10**, **11**, and **14**, only reductive trends were seen when LAT1 was inhibited (Figure S2). This is most likely due to their higher ability to utilize secondary transport mechanisms, such as OATPs, for their cellular uptake. Nevertheless, in the present study, the highest cellular uptake was observed with the ester compounds (**1**, **14**, **15**), which all were structurally relatively large (Mw. 393.44–498.55 g/mol) and lipophilic (clogP 1.04–1.50) molecules. However, as LAT1 is facilitating transporter carrying its substrates down their concentration gradient, and since these ester prodrugs were relatively rapidly bioconverted to their parent drugs within the cells, it can be speculated whether the bioconversion is the driving force to these compounds or ester prodrugs are just simply better substrates, e.g., LAT1 and OATPs. However, the peripheral or pre-mature bioconversion of the ester prodrugs needs to be optimized, or alternatively, peripheral enzyme inhibitors, such as carboxylesterase inhibitors, need to be utilized to ensure effective transport of LAT1-ester prodrugs into the brain [39]. Curiously, compound **4**, which is a pure D-amino acid form unlike the other studied compounds, was effectively transported into the cells, implying that LAT1 can carry not only L-amino mimetics but also D-amino mimetics into the cells.

### 2.4. LAT1-Utilizing Prodrugs Do Not Induce the Expression or Function of LAT1

Interestingly, according to the concentration-dependent uptake results (Table 3, Figure S1), some of the LAT1-utilizing compounds (**8**–**10** and **12**–**15**) had an autoactivated Eadie–Hofstee profile, meaning the uptake of these compounds was induced by themselves (homotropic positive cooperativity) [40]. To study this phenomenon in more detail, we pre-incubated BV2 cells with LAT1-utilizing compounds **1**–**15**, LAT1-substrate thyroxin (T_4_), known nuclear receptor inducible compounds, phenobarbital (constitutive androstane receptor, CAR), and dexamethasone (pregnane X receptor, PXR) (100 µM), as well as L-glutamine (intracellular substrate for LAT1 antiport) (2 mM) for 10 min or 3 h before measuring the cellular uptake of LAT1-substrate L-leucine (1–400 µM; 5 min). As seen in Figure S3, most of the LAT1-utilizing compounds did not induce the cellular uptake of L-Leu. Compounds **1** and **4**, as well as dexamethasone and phenobarbital, did not have any effect on the uptake, while compounds **3** and **7**–**15** and L-glutamine decreased the amount of L-Leu inside the cells, implying that they inhibited the L-Leu uptake even though the compounds were removed from the cells before adding L-Leu. The time-dependent uptake of compounds **3** and **7**–**15** varied a lot; thus, no common factor was found to explain these results. Most likely they were good LAT1-binders and slowly transported into the cells that affected the cellular uptake of L-Leu. Unlike the other compounds, compounds **2**, **5**, **6**, and T_4_ induced the uptake of L-Leu. The inducing effect was highest with compound **5**, both after short as well as longer pre-incubation (Figure S3). Curiously, these compounds were not the ones for which the autoactivation Eadie–Hofstee profile was observed and the cellular uptake of compound **5** was decreasing in the time-dependent uptake assay. Therefore, we postulate that the autoactivation profiles stem from the fact that there are not enough data points particularly in the linear part of the sigmoidal curve of the cellular uptake (the middle part in the concentration-dependent curve), in which two transport mechanisms overlap. This part of cellular uptake, in which two distinct transporters can carry the compound into the cells, creates the Eadie–Hofstee curve that resembles autoactivation or an inducible profile. For example, in the case of compound **8** (Figure S1), there are not enough data points for LAT1-mediated uptake; the lower affinity-higher capacity transport is clear, while the higher affinity-lower capacity transport has only two or three points. Contrarily, with compounds **10**, **13**, and **15** the situation is vice versa; the higher affinity–lower capacity transport is clear, while the lower affinity–higher capacity transport lacks data points. For the comparison, compounds **9**, **12**, and **14** have relatively large concentration areas in which two transport mechanisms are overlapping, and therefore, the Eadie–Hofstee profile is not able to clearly separate either of them. Thus, according to these results, the LAT1-utilizing compounds **1**-**15** were not able to upregulate the LAT1 function. Furthermore, we concluded that those compounds (compounds **2**, **5**, **6**, T_4_, and phenobarbital) that were able to induce the cellular uptake of L-Leu, most likely drove L-Leu to another higher capacity transporter. Curiously, it was also expected that LAT1 could be induced by a high concentration of intracellular L-glutamine, since it has been postulated that LAT1 has a higher affinity for intracellular amino acids compared to extracellular ones [6,7,27]. However, according to our results, L-glutamine was not able to induce L-Leu uptake; instead, it had a reducing effect (Figure S3).

**Figure 3:**
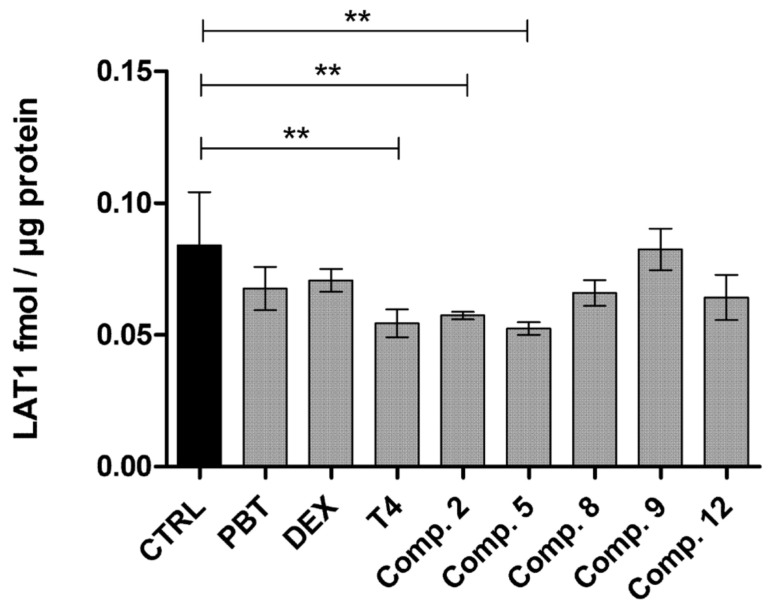
Quantitative protein levels of LAT1 normalized to a housekeeping protein Na+ /K+ATPase and the total amount of protein in the sample. The results are expressed as mean ± SD (*n* = 3) and the asterisks denote a statistically significant difference, ** *p* < 0.01, one-way ANOVA, followed by Tukey’s test. The abbreviation PBT denotes phenobarbital and DEX dexamethasone.

To confirm our conclusions, we also measured the LAT1 expression (normalized to plasma protein Na^+^K^+^ATPase) by a quantitative proteomic method with LC-MS. Thus, BV2 cells were incubated 24 h with selected compounds, including those that originally seemed to have autoactivated Eadie–Hofstee profile (compounds **8**, **9** and **12**) as well as those which were able to induce the cellular uptake of L-Leu (compounds **2**, **5**, and T_4_) and phenobarbital and dexamethasone, which did not have any effect on LAT1 function. As seen in Figure 3, only T_4_ and compounds **2** and **5** downregulated LAT1 expression on the plasma membrane compared to the untreated control sample, while phenobarbital, dexamethasone and compounds **8**, **9**, or **12** did not have any effect on LAT1 expression. Thus, it can be concluded the induced uptake, either L-leu or the compounds themselves, mainly occurred due to the secondary transport mechanism rather than regulation of LAT1.

### 2.5. LAT1-Utilizing Compounds Accumulates into Lysosomes, but Do Not Affect Cell Viability or Brain Amino Acid Homeostasis

**Figure 4:**
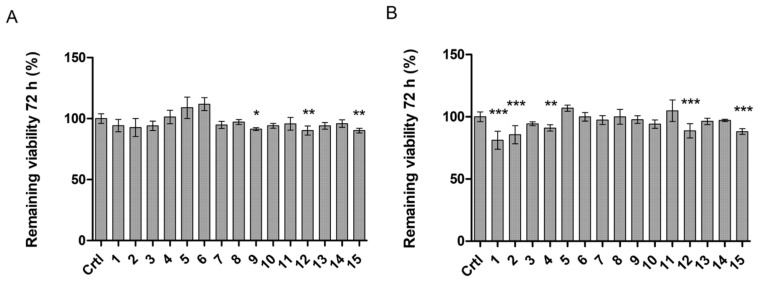
The remaining cell viability (%) after 72 h incubation of (**A**) 50 µM or (**B**) 100 µM LAT1-utilizing compounds **1**–**15** with immortalized mouse microglia (BV2 cells). The data are expressed as mean ± SD, *n* = 3–6 and asterisk denotes a statistically significant difference compared to the untreated control (crtl) cells (* *p* < 0.05, ** *p* < 0.01, *** *p* < 0.001), one-way ANOVA followed by Dunnett’s test).

To evaluate the ability of the studied LAT1-utilizing compounds (**1**–**15**) to affect the cell viability of immortalized mouse microglia, the compounds were incubated 72 h with the cells, after which the aerobic respiration of metabolically active cells was determined with Alamar blue (resazurin). With lower concentrations (<50 µM), none of the studied compounds affected the cell growth markedly, as the viability of all groups was over 90% compared to the control (Figure 4A). Only compounds **9**, **12**, and **15** showed minor effects on cell viability at 50 µM concentrations. In addition, with higher concentrations (>100 µM), compounds **1**, **2**, **4**, **12**, and **15** started to impact the cell viability (Figure 4B). Nevertheless, this reduction in cell growth was still minor (9–19% reduction) and the used concentrations were relatively high compared to the concentrations achieved with the in vivo situations. Therefore, it was concluded that these compounds do not affect the aerobic respiration of the cells. However, more advanced toxicity studies are required to confirm this observation.

**Figure 5:**
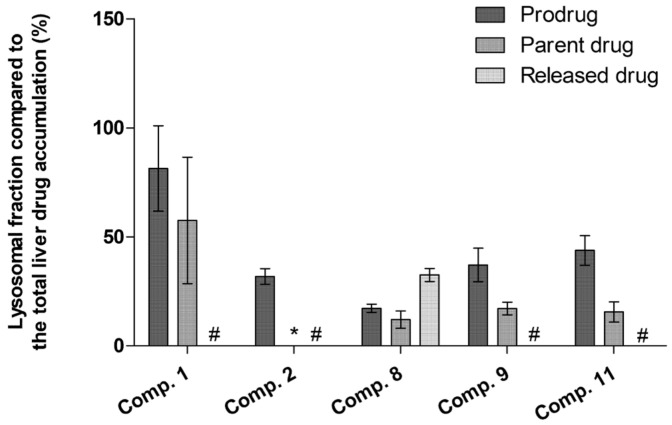
Liver lysosomal accumulation of prodrugs **1**, **2**, **8**, **9**, and **11** and their released parent drugs compared to the lysosomal accumulation of their parent drugs expressed as percentages (%) of total liver tissue accumulation. The liver samples were collected at the t_max_ (10–120 min) of each prodrug according to their pharmacokinetic evaluation [28,31,41]. An asterisk (*) denotes that no parent drug was detected (compound **2**) and hashtag (#) that no released parent drug was observed (all prodrugs except compound **8**).

In addition, to the systemic pharmacokinetic study that is usually carried out early in the preclinical (pro)drug development [28,30,31,41], it is very important to understand the intracellular kinetics of (pro)drugs in the target cells. We have previously shown that LAT1-utilizing compounds can be effectively uptaken into isolated lysosomes [32], since LAT1 is known to be localized in addition to the plasma membrane, also in lysosomal membranes [26]. In the present study, crude lysosomal fractions from the tissue homogenates of the mouse liver samples after a single i.p. dose of 25 µmol/kg of selected compounds (**1**, **2**, **8**–**11**) were isolated and the lysosomal accumulation was compared to the total tissue accumulation. Liver samples were selected for this experiment due to the relatively high accumulation of these LAT1-utilizing compounds into the liver and to keep the amount of lysosomal accumulation within the detection limits of the used mass spectrometric method. Curiously, these results clearly showed that all LAT1-utilizing compounds were readily accumulated into the crude lysosomal fraction; 17–81% from total liver tissue accumulation, and more effectively than their parent drugs (12–57%), which were not detected at all in some cases (Figure 5). However, these results need to be interpreted with caution, since only crude lysosomal fraction was isolated from the in vivo samples. Moreover, even though the lysosomes stayed intact during the sample preparation, it is acknowledged that compounds may re-distribute during the centrifugation steps. Therefore, to fully reveal the intracellular kinetics of LAT1-utilizing compounds, more sophisticated imaging methods with labelled compounds should be used.

**Figure 6:**
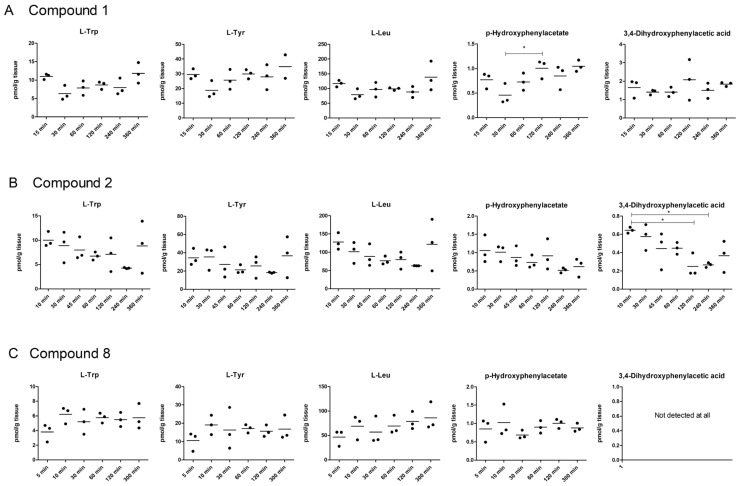

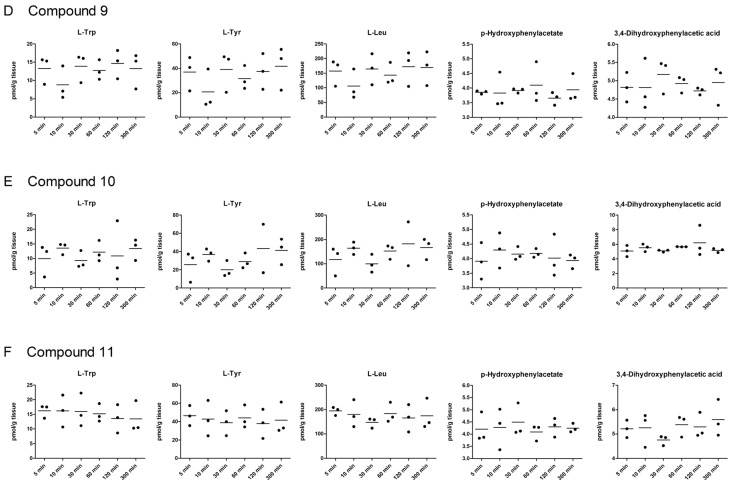
Brain amounts of amino acids L-Trp, L-Leu, L-Tyr and possible metabolites *p*-hydroxyphenylacetate and 3,4-dihydroxyphenylacetic acid of compounds **1** (**A**), **2** (**B**), **8** (**C**), **9** (**D**), **10** (**E**), and **11** (**F**) over 300–360 min period after a single dose of compound **1** (25 µmol/kg, i.p.) to mice. The data are presented as mean of each time point (*n* = 3) and an asterisk denotes a statistically significant difference compared to the first time point (5–15 min) (* *p* < 0.05, one-way ANOVA followed by Dunnett’s test).

As the main function of LAT1 at the BBB and parenchymal cells [19,20] is to ensure the essential amino acid supply to the neurons and glial cells, it is important to evaluate how LAT1-utilizing compounds affect the amino acid homeostasis in vivo, in the relevant tissues, such as the brain. In the present study, we evaluated how the LAT1-utilizing prodrug treatments affect the brain homeostasis of three amino acids, known to utilize mainly LAT1 for their brain uptake, L-Leu, L-Trp, and L-Tyr. Thus, the amount of the amino acids was analyzed from the mouse brain samples after a single i.p. dose of 25 µmol/kg of selected compounds (**1**, **2**, **8**–**11**) at different time points (5–360 min). None of the studied compounds affected the amino acid levels during the evaluation period; therefore it can be concluded that these compounds do not affect L-Leu-mTOR-mediated energy metabolism or L-Trp- or L-Tyr-mediated serotonin or dopamine synthesis, respectively (Figure 6). Most likely, there are high enough basal levels of essential amino acids in the brain, so that temporal inhibition for their entry across the BBB does not have a major effect on their homeostasis. Moreover, even though compound **2** was able to induce the uptake of LAT1-substrate and compounds **8**–**11** was, in turn, able to reduce the uptake of LAT1-probe-substrate on the plasma membrane in vitro (Figure S2), no major effects were seen in brain amino acids levels in vivo.

In addition to the above-mentioned amino acids, two L-Tyr metabolite levels, *p*-hydroxyphenylacetate and 3,4-dihydroxyphenylacetic acid, were followed. As seen in Figure 6, most of the compounds did not affect the L-Tyr metabolite levels. Curiously, with compound **8** no 3,4-dihydroxyphenylacetic acid was detected at all. However, this may be related to L-Tyr metabolism by this specific compound rather than its effects on the LAT1 function at the BBB. Only compound **2** showed a significant reduction of 3,4-dihydroxyphenylacetic acid levels at the time points of 2 and 4 h compared to the first time point (10 min). However, this effect on the L-Tyr metabolism most likely started to recover, as it can be indicated from the 6 h time point (Figure 6). Furthermore, compound **1** showed some fluctuating levels of *p*-hydroxyphenylacetate, particularly after 30 min of the bolus injection, when the brain *p*-hydroxyphenylacetate levels were significantly lower than in other time points. This reflects the phenomenon of relatively rapid brain uptake of compound **1** that can inhibit normal LAT1 function at the BBB only for a short time, which is then normalized as the amount of compound **1** decreases in the systemic circulation. Taking all these results together, it was concluded that it is very important to evaluate how the LAT1-utilizing compounds are distributed within the cells, and how they affect cellular viability, as well as the normal functions of LAT1.

## 3. Discussion

Despite the attractiveness of transporters being druggable targets or site-selective carriers, there is a lack of proper tools and methods to study these proteins’ biological role and usefulness in drug development [42]. Moreover, the current methods that are used for transporter studies are based on enzyme kinetics, which is not optimal [43]. In the present study, we evaluated the utilization of LAT1 by several assays, which can vary a lot between the laboratories. When using a *cis*-inhibition assay, the selection of radiolabelled substrate does not seem to have a significant effect on the final conclusions (Table 1). Since this method measures only the competitiveness of a substrate to inhibit the cell entry of a radio-probe, it cannot exclude the involvement of other transport mechanisms in the radio-ligand transport process; thus, the results need to be interpreted very carefully. Moreover, with this method, it cannot be determined whether the studied compound is a substrate (carried across the membrane via the transporter) or an inhibitor (binding only to the transporter). Therefore, not even the kinetic analysis with Hanes-Woolf plots can explain the differences between the compounds in the transport efficiency without additional methods (Table 2), such as computational molecular dynamics or direct cellular uptake studies. Nevertheless, the former mentioned method together with *trans*-stimulation assay (in which LAT1-expressing cells are loaded with radiolabelled LAT1-probe substrate) are widely accepted, due to their easiness and rapidity to produce the results, as well as due to their cheaper price compared to the exact uptake amount and rate measured by, e.g., liquid chromatography-mass spectrometric (LC-MS) methods.

With direct cellular uptake assay, e.g., by LC-MS, both time and concentration dependency can be explored to reveal the transport efficacy across the plasma membrane via LAT1. In addition, by using linear post-transformation methods, such as Eadie–Hofstee plotting, the possible secondary transport mechanisms can be identified, if native (non-transfected) cells are used in the assay (Table 3, Figure S1). However, computational methods combined with experimental data can be very helpful also to gather information on the interactions of the compounds during the transport process [36]. LAT1 is known to have a so-called “rocking-bundle alternating-access mechanism” and therefore, interactions of ligands can promote the conformational changes that define the extracellular and intracellular gates [5,44]. These changes enable the movements of the ligand, as well as the anti-transported amino acid in the cavity. According to the present study, amino acid prodrugs with ester bonds may be better LAT1-substrates than their corresponding amide prodrugs. Esters are relatively rapidly bioconverted to their parent drugs, which may be a driving force for facilitated diffusion but may also predispose for premature bioconversion during first-pass metabolism. However, due to the parent drug moiety, the uptake rate via LAT1 can vary a lot among the studied compounds (Figure 2 and Figure S1), implying that the transport process through the LAT1 cavity can be either slow or fast, depending on the structural properties of the whole compound and how they induce the “rocking-bundle alternating-access mechanism” and conformational changes in the LAT1 cavity.

Moreover, it is also very important to study how LAT1-utilizing compounds behave intracellularly (how they are distributed inside the cells), how they affect cellular viability, or the expression or function of LAT1 on the plasma membrane. In this study, we explored how the LAT1-utilizing compounds can affect LAT1 function and quantitative expression, since according to the Eadie–Hofstee plotting, some of the uptake curves implied homotropic positive cooperativity, the so-called “autoactivation” of the transport mechanism. However, it was found that if another transport mechanism is involved in addition to LAT1 to the transport process of the studied compound and there are not enough data points, one may obtain a sigmoidal curve in the Eadie–Hofstee plot, indicating a possible autoactivation of the transporter (Figure S1). According to the results presented here, none of the studied LAT1-utilizing compounds were able to induce the function or expression of LAT1 (Figure S3 and Figure 3). However, the effects of possible metabolites produced from the studied compounds need to be evaluated more thoroughly in the future.

It is also well known that, intracellularly, LAT1 is expressed on the lysosomal membranes of cells. Thus, it is highly likely that LAT1-utilizing compounds have also a tendency to be accumulated into the lysosomes. However, this has not been studied extensively in the past, although it may have a significant impact on the efficacy and safety profile of the studied compound. In the present study, all the studied compounds were found to be delivered extensively into the lysosomes, which may limit their use, as their final targets are in the cytosolic site (soluble and membrane-bound cyclooxygenase, reactive oxygen species (ROS)) or secreted gigantosomes (perforin) (Figure 5). Therefore, intracellular kinetics of LAT1-utilizing compounds should be studied in the very early phase of the drug development process. Moreover, the effects that LAT1-utilizing compounds may have on normal functions, such as amino acid balance in the brain, should be considered as early as possible. Even though the results presented herein pointed out that exogenous LAT1-utilizing compounds that can compete temporarily with natural substrates for cell entry via LAT1, do not affect the amino acid brain homeostasis and cell viability (Figure 4 and Figure 6), it may be compound-specific; therefore, these aspects should also be evaluated very carefully in the early stage of the drug development.

## 4. Materials and Methods

### 4.1. Materials

All reagents and solvents used in analytical studies were commercial and high purity of analytical grade or ultra-gradient HPLC-grade and purchased either from Merck KGaA/Millipore-Sigma (Munich, Germany), J.T. Baker (Deventer, The Netherlands), VWR International, LCC (Radnor, PA, USA), Gibco, (ThermoFisher Scientific, Waltham, MA, USA), or EuroClone S.p.A. (Pero, Italy). Water was purified using a Milli-Q Gradient system (Millipore, Milford, MA, USA). The studied LAT1-utilizing prodrugs have been synthesized in-house; their structural characterization (^1^H NMR, ^13^C NMR, LC-MS) and over 95% purity (elemental analysis) have been confirmed in our earlier publications; the studied prodrugs were made of perforin inhibitor (**1**) [32], ketoprofen (**2**–**4, 14**) [28,29,33] and ferulic acid (**5**–**7**) [30], salicylic acid (**8**) [31], flurbiprofen (**9**, **12**) [31,33], ibuprofen (**10**, **13**) [31,33] and naproxen (**11**, **15**) [31,33]. The molecular masses and logP values of the studied compounds were calculated by using ChemDraw Professional (v16.0.1.4 (77); PerkinElmer Informatics, Inc., Waltham, MA, USA) software.

### 4.2. Cell Cultures

Immortalized microglia (BV2) were selected for this study due to their relatively high expression of LAT1 (3.99 ± 0.91 fmol/µg protein) [20]. The cells were cultured in RPMI-1640 medium containing L-glutamine (2.0 mM), heat-inactivated fetal bovine serum (10%), penicillin (50 U/mL) and streptomycin (50 µg/mL) solution. The cells (passages of 8–15) were seeded at the density of 10^5^ cells/well onto 24-well plates one day before the experiments. All the studies were carried out as three biological replicates from the same cell passage. The function of LAT1 was followed between the used cell passages with a known LAT1 probe substrate, [^14^C]-L-leucine (PerkinElmer, Waltham, MA, USA). No changes in LAT1 function were noticed during this study (V_max_ 0.84 ± 0.12 nmol/min/mg protein; K_m_ 85.8 ± 19.4 µM).

### 4.3. Cis-Inhibition Studies (Ability of Compounds Bind to LAT1 and Inhibit Amino Acid Uptake)

After removal of the culture medium, the BV2 cells were carefully washed with pre-warmed HBSS (Hank’s balanced salt solution) containing 125.0 mM choline chloride, 4.8 mM KCl, 1.2 mM MgSO_4_, 1.2 mM KH_2_PO_4_, 1.3 mM CaCl_2_, 5.6 mM glucose, and 25.0 mM HEPES (pH 7.4 adjusted with 1 M NaOH). Pre-incubation was done with 500 μL of pre-warmed HBSS at 37 °C for 10 min before adding substrates (250 μL in HBSS) for the uptake experiments.

The ability of compounds **1**–**15** to inhibit the uptake of a known LAT1 substrate [^14^C]-L-leucine was then evaluated by incubating the cells at 37 °C for 5 min in uptake buffer pH 7.4 (250 μL) containing 0.2 µCi/mL of [^14^C]-L-leucine, or 2 µCi/mL of [^3^H]-L-methionine, [^3^H]-L-tryptophan or [^3^H]-L-kynurenine (PerkinElmer, Waltham, MA, USA) and 0.1–1000 μM of the studied compound (or HBSS as blank). After incubation, the uptake was stopped by adding 250 µL of ice-cold HBSS, and the cells were washed two times with ice-cold HBSS. The cells were then lysed with 500 μL of NaOH (0.1 M) for 60 min and the lysate was mixed with 3.5 mL of Emulsifier safe cocktail (Ultima Gold, PerkinElmer, Waltham, MA, USA). The radioactivity in the cells was measured by liquid scintillation counting (MicroBeta^2^ counter, PerkinElmer Waltham, MA, USA). Half of the maximum inhibitory concentration (IC_50_) values were calculated by nonlinear regression analysis (fitting the curve to log (concentration) vs. remaining normalized viability).

To evaluate the inhibition type of studied compounds, the BV2 cells were incubated with 0.010–0.20 μM [^14^C]-L-leucine together with the studied compound. The concentration of compounds was selected according to the compound-specific IC_50_ values. The experiment was then carried out as described for the [^14^C]-L-leucine uptake above and the radioactivity was measured by liquid scintillation counting. Hanes-Woolf plots were created by setting the ratio of initial substrate concentration to the reaction velocity ([S]/v) against [^14^C]-L-leucine concentration (µM). The linear regression was used to calculate K_m_ value (negative value of x-intercept) and V_max_ value (1/slope) for [^14^C]-L-leucine.

### 4.4. Time-Dependent Cellular Uptake of Compounds

For the time-dependent cellular uptake experiments, the BV2 cells on 24-well plates were incubated with studied compounds (100 μM) in a pre-warmed HBSS buffer (250 μL) at 37 °C for 1–60 min. Subsequently, the cells were washed three times with an ice-cold HBSS and lysed with 250 μL of NaOH (0.1 M) for 60 min. The lysates were diluted with acetonitrile (ACN) including the selected internal standard (diclofenac to all compounds) with a ratio of 1:3 and centrifuged at 10,000× *g* for 10 min. The samples were analyzed by the liquid chromatography-tandem mass spectrometric (LC-MS/MS) methods as described below. The concentrations of each compound in cell lysates were calculated from the standard curve that was prepared by spiking known amounts of compounds to ACN including the selected internal standard and then normalized with protein concentration. The protein concentrations on each plate were determined as a mean of three samples by Bio-Rad Protein Assay, based on the Bradford dye-binding method, using bovine serum albumin (BSA) as a standard protein and measuring the absorbance (595 nm) by multiplate reader (EnVision, Perkin Elmer, Inc., Waltham, MA, USA).

### 4.5. Concentration-Dependent Cellular Uptake of Compounds

For the concentration-dependent cellular uptake experiments, the BV2 cells on 24-well plates were incubated with studied compounds (1–200 μM) in pre-warmed HBSS buffer (250 μL) at 37 °C for 30 min. The experiment was then carried out as described for the time-dependent uptake above and the concentration of studied compounds in the cell lysate was analyzed by the LC-MS/MS methods described above and the results were normalized to the total protein concentration. The distinct transport mechanisms were separated from the Michaelis-Menten kinetics by using Eadie–Hofstee plot.

### 4.6. LAT1-Mediated Cellular Uptake of Compounds

The competitive uptake in the presence of LAT1-inhibitor (KMH-233) [20] was carried out as described above with HBSS buffer solution containing 100 µM of the studied compounds. The cells were pre-incubated with 100 µM LAT1-inhibitor for 10 min and the incubation mixture was removed before adding the studied compound and LAT1-inhibitor on the cells. The competitive uptake (30 min) with the inhibitor was then carried out as the normal uptake described above. The amounts of studied compounds were analyzed by the LC-MS method and calculated from the spiked standard curve and normalized with the protein concentrations.

### 4.7. Liquid Chromatography-Tandem Mass Spectrometric (LC-MS/MS) Methods

The studied compounds (**1**–**15**) were analyzed by using Agilent 1200 Series Rapid Resolution LC System together with an Agilent 6410 Triple Quadrupole Mass Spectrometer equipped with an electrospray ionization source (Agilent Technologies, Santa Clara, CA, USA). The chromatographic separations of analytes were achieved by using a reversed-phase Zobrax SB-C18 column (50 mm × 2.1 mm, 1.8 μm; Agilent Technologies, Santa Clara, CA, USA) by eluting the samples with 0.1% formic acid in water (A) and 0.1% formic in ACN (B). Labetalol and diclofenac were used as internal standards (ISTD) for the prodrugs and parent drugs, respectively. The transitions used for the quantification at the positive ion mode and chromatographic conditions of each compound and their parent drugs are listed in Table S1. The methods of compounds **1**–**11** have been reported earlier [28,30,31,41], while the methods of compounds **12**–**15** are reported herein for the first time.

The LC-MS/MS methods were validated according to the bioanalytical method validation guidance for the industry. The methods were linear over the range 1.0–100 nM and they were specific (no interference was observed), and within-run accuracy of the quality control samples were ± 15% of the nominal concentrations and their precision RSD < ±15%.

### 4.8. Concentration-Dependent Cellular Uptake of L-Leucine in the Presence of the Compounds

The ability of compounds **1**–**15** to affect the uptake of a known LAT1 substrate L-leucine was evaluated by pre-incubating the BV2 cells at 37 °C for 10 min or 3 h with uptake buffer pH 7.4 (250 μL) containing 100 µM of compounds **1**–**15** or thyroxin (T_4_), or known nuclear receptor inducible compounds, phenobarbital (constitutive androstane receptor, CAR), and dexamethasone (pregnane X receptor, PXR) as controls. Afterward, the pre-incubation solution was removed and the cellular uptake of 1–400 µM L-leucine containing 0.76 μM (0.25 mCi/mL) of [^14^C]-L-leucine (PerkinElmer, Waltham, MA, USA) in 250 μL of HBSS buffer was measured after 5 min incubation at 37 °C. After incubation, the reaction was stopped by adding 250 µL of ice-cold HBSS, and the cells were washed two times with ice-cold HBSS. The cells were then lysed with 500 μL of 0.1 M NaOH (60 min) and the lysate was mixed with 3.5 mL of Emulsifier safe cocktail (Ultima Gold, PerkinElmer, Waltham, MA, USA). The radioactivity in the cells was measured by liquid scintillation counting (MicroBeta^2^ counter, PerkinElmer Waltham, MA, USA). The results were multiplied by the amount of un-labeled proportion of L-leucine in each sample.

### 4.9. Quantitative Expression of LAT1 on the BV2 Plasma Membrane

The protein expression levels of LAT1, in the plasma membrane fractions of BV2 cells were quantified using multiplexed MRM analysis according to the protocol described by Uchida et al. [45]. First, the crude membrane fractions were isolated from three distinct sets of cell culture plates using Membrane Protein Extraction Kit (BioVision Incorporated, Milpitas, CA, USA) according to the manufacturer’s instructions. The protein content for each fraction was measured by Bio-Rad Protein Assay, based on the Bradford dye-binding method (EnVision, Perkin Elmer, Inc., Waltham, MA, USA). A total amount of 50 µg protein from each fraction was solubilized/denatured in 7 M guanidine hydrochloride, 0.5 M Tris-HCl and 10 mM EDTA-Na. The proteins were then reduced by dithiothreitol (1:50, *w*/*w*) and *S*-carboxymethylated by iodoacetamide (1:20, *w*/*w*). The alkylated proteins were precipitated by methanol/chloroform/water (4:1:3) and centrifuged at 18,000× *g* for 5 min at 4 °C. The pellet was resuspended in 6 M urea and mixed for 10 min at room temperature before the dilution with 0.1 M Tris-HCl (pH 8.5) to a final concentration of 1.2 M urea, and dissolved completely by intermittent sonication (Branson 3510, Danbury, CT, USA). The dissolved proteins were first digested with LysC (1/100, *w*/*w*) and 0.05% ProteaseMax (Promega Biotech AB, Nacka, Sweden) for 3 h at room temperature. Then, the samples were spiked with 10 µL (30 fmol) of the labelled peptides for absolute quantification (JPT Peptide Technologies GmbH, Berlin, Germany) (Table S2). The samples were incubated with (1/100, *w*/*w*) TPCK-Trypsin (Promega Biotech AB, Nacka, Sweden) for 18 h at 37 °C. The tryptic digestion was then quenched by adding 40 µL of 5% formic acid. The samples were then centrifuged at 18,000× *g* for 5 min at 4 °C, and the supernatants were transferred to vials for analysis.

The digested peptides were analyzed using an ultra-performance liquid chromatography system coupled with a triple quadrupole mass spectrometer with a heated electrospray ionization source in the positive mode (UPLC 1290 and MSD 6495, Agilent Technologies, Santa Clara, CA, USA). A total amount of 20 µL of the digested peptides (10 µg) was separated using AdvanceBio Peptide Map 2.1 × 250 mm, 2.7 μm column (Agilent Technologies, Santa Clara, CA, USA) and LC eluents of 0.1% formic acid in water (A) and acetonitrile (B). The peptides were eluted following a constant flow rate of 0.3 mL/min and a gradient of 2–7% B for 2 min, followed by 7–30% B for 48 min, 30–45% B for 3 min, and 45–80% B for 2.5 min before re-equilibrating the column again for 4.5 min. LAT1 was quantified based on the ratio between the light and heavy standard peptides, as described previously (Table S2) [31]. Data was acquired using Agilent MassHunter Workstation Acquisition (Agilent Technologies, Data Acquisition for Triple Quadrupole, version B.03.01) and processed by using Skyline software (version 20.1). The results were normalized to a housekeeping protein Na+/K+ATPase and expressed as fmol/µg of the total amount of protein in the samples.

### 4.10. Cellular Viability

Immortalized microglia (BV2 cells) were seeded at the density of 10 × 10^3^ cells/well onto collagen-coated 96-well plates. The cells were used for the proliferation experiments one day after seeding. The studied compounds (50 and 100 µM) were added into the growth medium and incubated for 3 days (the medium was replaced with a fresh one with studied compounds after daily measurements). Each day the cell viability was determined by the resazurin cell proliferation kit (MilliporeSigma, St. Louis, MO, USA), which is directly proportional to aerobic respiration and cellular metabolism of cells. The samples were measured fluorometrically by monitoring the increase in fluorescence at λ_ex_ 560 nm and λ_em_ 590 nm with the Envision plate reader (EnVision, Perkin Elmer, Inc., Waltham, MA, USA). The cell viability was also followed by visualizing the wells with microscopy. The ability of compounds to inhibit the viability of the cells was expressed as percentages (%) compared to the untreated controls.

### 4.11. Animals

Adult male mice weighing 25 ± 5 g were supplied by Envigo (Venray, Netherlands). Mice were housed in stainless steel cages on a 12 h light (07:00–19:00) and 12 h dark (19:00–07:00) cycle at an ambient temperature of 22 ± 1 °C with a relative humidity of 50–60%. All experiments were carried out during the light phase. Tap water and food pellets (Lactamin R36; Lactamin AB, Södertälje, Sweden) were available ad libitum. A dose of 25 µmol/kg of compound **1**, **2**, or **8**–**11** was given as a bolus injection (i.p.) to mice. The mice were decapitated at selected time points between (10–360 min) and brain tissues were collected for sample preparation to be analyzed by liquid chromatography-mass spectrometric (LC-MS/MS) analysis.

### 4.12. Lysosomal Accumulation of LAT1-Utilizing Compounds

The liver lysosomal samples (10–120 min; *t_max_*) [28,31,41] were prepared by homogenizing the pre-weigh tissues with ice-cold 0.25 M sucrose (1:3). Then, 100 µL of the homogenate was taken, and the proteins were precipitated with 100 µL of ACN containing the internal standard (labetalol). Samples were vortexed and centrifuged at 10,000× *g* at 4 °C for 10 min and injected to LC-MS/MS to quantify the total drug amount in tissue as a control to lysosomal samples. To separate crude lysosomal fraction, 500 µL of liver homogenate (1:3) was centrifuged at 1000× *g* at 4 °C for 10 min. The supernatants were then transferred to other microcentrifuge tubes and re-centrifuged at 15,000× *g* at 4 °C for 20 min. The pellets were collected and resuspended with ice-cold 0.25 M sucrose and homogenized gently. The lysosomes (100 µL) were then lysed and precipitated with ice-cold ACN (100 µL) containing the internal standard (labetalol), vortexed, and centrifuged at 10,000× *g* at 4 °C for 10 min. The concentration of compounds in the crude lysosomal fractions were then analyzed with LC-MS/MS. Both the total liver tissue accumulation as well as the lysosomal fraction (including the possibly released parent drugs) were calculated from the standard curve that was prepared by spiking known amounts of compounds to ACN, including the selected internal standard. The final results are represented as lysosomal amount compared to the total homogenate amount and expressed as percentages (%).

### 4.13. Brain Amino Acid Homeostasis

Brain samples were prepared similarly as described above by homogenizing the pre-weigh tissues with ultrapure water (1:3). Subsequently, 100 µL of the homogenates was taken, and the proteins were precipitated with 300 µL of ACN containing the internal standard (labetalol).

Samples were vortexed and centrifuged at 10,000× *g* at 4 °C for 10 min. 200 µL of supernatant was mixed with 100 µL of ultrapure water and injected to LC-MS/MS equipment described above. The amounts of three known LAT1-utilizing amino acids, L-Leu, L-Tyr, and L-Trp, and two L-Tyr metabolites, namely *p*-hydroxyphenylacetate and 3,4-dihydroxyphenylacetic acid, were quantified with a method described earlier [46]. Briefly, reversed-phase Zobrax SB-C18 column (50 mm × 2.1 mm, 1.8 μm; Agilent Technologies, Santa Clara, CA, USA) was used at a flow rate 0.3 mL/min with gradient elution of eluents consisting of 20 mM ammonium formate in H_2_O:ACN (1:1; A) and 20 mM ammonium formate in H_2_O:ACN (1:9; B), pH 3. Mass spectrometric detection was performed with multiple reaction monitoring at the positive mode with the following transitions: 182.0→164.9 for L-Tyr, 132.1→86.1 for L-Leu, 205.1→188 for L-Trp, 181.0→119.0 for *p*-hydroxyphenylacetate, 167.0→123.0 for 3,4-dihydroxyphenylacetic acid, and 329.0→294.0 for labetalol. Data were acquired using the Agilent MassHunter Workstation Acquisition software (Data Acquisition for Triple Quadrupole Mass Spectrometer, version B.03.01) and processed and analyzed with Quantitative Analysis (B.04.00) software. The lower limit of quantification (LLOQ) for the amino acids in tissue samples was 0.1 nM for L-Leu, L-Tyr, and L-Trp and 1 nM for *p*-hydroxyphenylacetate and 3,4-dihydroxyphenylacetic acid. The methods were linear, selective, accurate, and precise over the calibration range of 0.25–2500 nM. The concentrations of the amino acids and their metabolites were calculated from the standard curve that was prepared by spiking known amounts of compounds to ACN including the selected internal standard.

### 4.14. Data Analysis

All statistical analyses, including Michaelis–Menten and Eadie–Hofstee and Hanes-Woolf analyses, were performed using GraphPad Prism v5.03 software (GraphPad Software, San Diego, CA, USA). Statistical differences between groups were tested using one-way ANOVA, followed by a two-tailed Tukey’s multiple comparison test and presented as mean ± SD, with significant difference denoted by * *p* < 0.05, ** *p* < 0.01, *** *p* < 0.001.

## 5. Conclusions

In conclusion, the detailed understanding of transporter-mediated drug delivery requires a wide variety of different methods and expertise that may not be found in a single research group. Nevertheless, most of the currently used methods are derived from enzyme kinetics and thus are suboptimal for transporter kinetics. This may limit the success of developing effective and safe transporter-utilizing drug therapies. Moreover, LAT1 has been extensively studied for decades; therefore, more efforts should also be paid on other transport proteins, especially those that are regarded as orphan ones.

## Figures and Tables

**Table 1:** The ability of LAT1-utilizing compounds (**1**–**15**) to bind LAT1 and inhibit cellular amino acid uptake into immortalized mouse microglia (BV2 cells) presented as half-maximal inhibitory concentrations (IC_50_ values) of selected probe substrates ([14C]-L-Leu, [3H]-L-Met, [3H]-L-Trp, [3H]-L-Kynu) uptake. The data are presented as mean ± SD, *n* = 3–4.

Compound	IC_50_ (µM)			
	[^14^C]-L-Leu	[^3^H]-L-Met	[^3^H]-L-Trp	[^3^H]-L-Kynu
**1**	6.8 ± 1.4	8.2 ± 1.7	6.7 ± 1.6	0.7 ± 0.1
**2**	2.8 ± 1.5	3.3 ± 1.5	9.9 ± 1.3	4.3 ± 1.3
**3**	26.0 ± 1.1	17.1 ± 1.9	39.2 ± 1.5	11.9 ± 1.3
**4**	15.0 ± 1.3	26.1 ± 1.4	38.8 ± 3.1	35.3 ± 1.2
**5**	>100	>100	>100	43.3 ± 1.1
**6**	>100	>100	>100	54.3 ± 1.3
**7**	37.6 ± 1.8	13.6 ± 1.8	19.8 ± 1.3	96.8 ± 1.2
**8**	18.9 ± 1.6 ^1^	12.0 ± 1.4	24.5 ± 2.0	18.1 ± 1.2
**9**	6.0 ± 1.5 ^1^	1.7 ± 1.8	15.9 ± 7.8	4.7 ± 1.2
**10**	13.0 ± 1.2 ^1^	9.6 ± 1.6	43.4 ± 1.5	7.7 ± 1.1
**11**	9.0 ± 1.1 ^1^	10.4 ± 1.5	14.0 ± 1.2	1.8 ± 1.2
**12**	4.2 ± 1.2 ^1^	4.3 ± 1.7	6.3 ± 1.3	3.0 ± 1.5
**13**	5.3 ± 1.2 ^1^	9.8 ± 1.4	36.3 ± 1.2	9.7 ± 1.2
**14**	52.9 ± 1.3 ^1^	32.0 ± 1.4	37.6 ± 1.7	91.5 ± 1.2
**15**	10.0 ± 1.4 ^1^	25.3 ± 1.6	11.1 ± 1.2	3.1 ± 1.3

^1^ Published in either in [31] or [33].

**Table 2:** Kinetic parameters (K_m_ and V_max_) of LAT1-mediated uptake inhibition of the probe substrate [^14^C]-L-Leu (0.010–0.20 µM) and the concluded inhibition type of LAT1-utilizing compounds **1**–**15** studied with immortalized mouse microglia (BV2). The data are presented as mean ± SD, *n* = 3–4.

Compound	IC_50_ (µM)	K_m_ (µM)	V_max_ (nmol/min/mg)	Inhibition Type
L-Leu (Crtl)	-	0.115 ± 0.007	3.403 ±0.055	
**1**	7	0.192 ± 0.0127	1.792 ± 0.106	Mixed type
**2**	3	0.202 ± 0.008	2.759 ± 0.066	Mixed type
**3**	10	0.109 ± 0.004	2.851 ± 0.035	Non-competitive
**4**	15	0.131 ± 0.020	1.599 ± 0.170	Non-competitive
**5**	9	0.057 ± 0.007	1.910 ± 0.057	Un-competitive
**6**	5	0.113 ± 0.009	3.224 ± 0.077	Non-competitive
**7**	38	0.267 ± 0.015	2.780 ± 0.126	Mixed type
**8**	19	0.071 ± 0.009	1.644 ± 0.073	Un-competitive
**9**	6	0.118 ± 0.007	2.422 ± 0.061	Non-competitive
**10**	13	0.171 ± 0.008	2.151 ± 0.066	Mixed type
**11**	10	0.098 ± 0.015	1.515 ± 0.125	Non-competitive
**12**	4	0.171 ± 0.026	1.513 ± 0.218	Mixed type
**13**	5	0.177 ± 0.028	2.186 ± 0.231	Mixed type
**14**	53	0.417 ± 0.032	2.622 ± 0.268	Mixed type
**15**	10	0.106 ± 0.012	1.448 ± 0.099	Non-competitive

**Table 3:** Michaelis-Menten kinetic parameters (V_max_, K_m_, and CL_int_) calculated from the Eadie–Hofstee plot analysis of transporter-mediated cellular uptake of studied compounds **1**–**15** (1–200 µM). The data are presented as mean ± SD, *n* = 3, and n.d. denotes a possible autoactivation mechanism.

Comp.	Transport Type 1 (LAT1)			Transport Type 2		
	V_max_ (pmol/mg*min)	K_m_ (µM)	CL_int_ (µL/min*mg)	V_max_ (pmol/mg*min)	K_m_ (µM)	CL_int_ (µL/min*mg)
**1**	750 ± 435	116 ± 72		5067 ± 327	347 ± 26	
**2**	0.09 ± 0.01	0.8 ± 0.1	0.1	0.88 ± 0.16	199 ± 50	0.004
**3**	3.7 ± 0.5	1.9 ± 0.3	1.9	49 ± 9	148 ± 41	0.33
**4**	88 ± 9	41 ± 6	2.2	231 ± 40	159 ± 45	1.45
**5**	0.11 ± 0.01	2.1 ± 0.4	0.05	0.27 ± 0.02	77 ± 13	0.004
**6**	0.11 ± 0.01	3.6 ± 0.4	0.03	0.26 ± 0.02	78 ± 16	0.003
**7**	0.09 ± 0.03	0.3 ± 0.1	0.3	0.21 ± 0.02	62 ± 14	0.003
**8**	n.d.	n.d.	n.d.	185 ± 10	41 ± 5	4.46
**9**	n.d.	n.d.	n.d.	-	-	-
**10**	205 ± 41	22 ± 6	9.5	n.d.	n.d.	n.d.
**11**	53 ± 8	2.6 ± 1.2	44.2	-	-	-
**12**	n.d.	n.d.	n.d.	-	-	-
**13**	63 ± 8	8.2 ± 1.8	7.7	n.d.	n.d.	n.d.
**14**	n.d.	n.d.	n.d.	-	-	-
**15**	280 ± 65	9.3 ± 2.7	30.1	n.d.	n.d.	n.d.

## Data Availability

Data sharing is not applicable to this article.

## Supplementary Materials

Figure S1: Cellular uptake of compounds **1**–**15** into immortalized microglia (BV2 cells), Figure S2: Cellular uptake of compounds **1**–**15** in the presence of LAT1-inhibitor, Figure S3: Cellular uptake of [14C]-L-leucine after incubation of compounds **1**–**15**, Table S1: LC-MS/MS ionization parameters of compounds **1**–**15**, Table S2: LC-MS/MS proteomic transitions for LAT1 peptides.

## Sample Availability

Samples of the compounds are available from the authors.
