# Supplementary material for: Comparison of Experimental Strategies to Study l-Type Amino Acid Transporter 1 (LAT1) Utilization by Ligands
Source: Molecules. 2021 Dec 22;27(1):37. doi: 10.3390/molecules27010037 (PMC8746705; doi:10.3390/molecules27010037)
Supplement: Supplementary file 1 [file molecules-27-00037-s001.zip › molecules-1473367-supplementary.pdf]

Supporting Information

Best Experimental Strategies to Study L-Type Amino Acid Transporter 1 (LAT1) Utilization by Ligands

Johanna Huttunen[1], Mahmoud Agami[1], Janne Tampio[1], Ahmed Montaser[1], Kristiina M. Huttunen[1,]*

[1] School of Pharmacy, Faculty of Health Sciences, University of Eastern Finland,

P.O. Box 1627, FI-70211 Kuopio, Finland

* Corresponding author, E-mail: kristiina.huttunen@uef.fi

Contents

1. *LC-MS/MS ionization parameters of compounds **1-15*** 2
2. *LC-MS/MS proteomic transitions for LAT1 peptides* 3
3. *Cellular uptake of compounds **1-15** into immortalized microglia (BV2 cells)*.................. 3
4. *Cellular uptake of compounds **1-15** in the absence and presence of LAT1-inhibitor* 6
5. *Cellular uptake of [^{14}C]-L-leucine after incubation of compounds **1-15*** 7

*1. LC-MS/MS ionization parameters of compounds **1-15***

Table S1. Ionization parameters for the LC-MS/MS methods of the studied compounds.

**Compound**	**Precursor ion**	**Product ion(s)**	**Fragmentor Voltage (V)**	**Collision Energy (V)**	**Flow rate (mL/min)**	**Gradient (A:B)**	**LLOQ (nM)**
**1 → 1a***	514 (322.8)	301 (203.7)	120 (140)	46 (34)	0.2	90:10 →10:90	0.5 (10)
**2**	417	135	70	30	0.4	80:20 →20:80	0.05
**3**	417	135	60	20	0.4	80:20 →20:80	0.05
**4 → KPF***	255	209	100	10	0.4	80:20 →20:80	0.1
**5**	371	177	60	16	0.3	90:10 →10:90	0.05
**6**	357	311	40	6	0.3	90:10 →10:90	0.05
**7 → FA***	358 (193)	176 (134)	60 (100)	16 (16)	0.3	90:10 →10:90	0.05 (1.0)
**8**	301	181	100	11	0.3	95:5 →5:95	3.0
**9**	407	361	170	13	0.3	95:5 →5:95	1.0
**10**	369	323, 161	140	13	0.3	95:5 →5:95	1.0
**11**	393	347, 185	150	15	0.3	95:5 →5:95	1.0
**12 → FLB***	408 (243)	362 (199)	175 (380)	15 (10)	0.5	95:5 →5:95	2.5 (2.5)
**13 → IBU***	370 (205)	324, 161 (161)	150 (380)	15, 19 (5)	0.5	95:5 →5:95	2.5 (2.5)
**14 → KPF***	255	209	100	10	0.4	80:20 →20:80	0.1
**15 → NPX***	394 (229)	348, 185 (170)	150 (380)	15, 19 (10)	0.5	95:5 →5:95	2.5 (2.5)
Diclofenac (ISTD)	294	250	50	3	-	-	-
Labetalol (ISTD)	329	294, 162	70	10	-	-	-

* Compounds **4** and **14** were fully converted into ketoprofen (KPF), and compound **1** partly to its investigational perforin inhibitor **1a** and therefore, their transitions with a positive ion mode are reported only as KPF or as a prodrug and parent drug in parentheses. Compounds **7**, **12**, **13**, and **15** were also partly converted to their parent drugs (ferulic acid (FA), flurbiprofen (FLB), ibuprofen (IBU), and naproxen (NPX), respectively), whose transitions with a negative ion mode are reported below the studied LAT1-utilizing compounds in parentheses.

2. *LC-MS/MS proteomic transitions for LAT1 peptides*

Table S2. SRM/MRM transitions for absolute quantitative proteomics.

Protein	St/IS	Unique amino acid sequence	Retention time (min)	Transition number	MRM transitions (m/z)	
					Precursor ion (Q1)	*Product inos (Q3)*
LAT1	St	VQDAFAAAK	13.7	1	460.7	821.4
				2		578.3
				3		507.3
	IS	VQDAFAAA**K***	13.7	1	464.8	829.4
				2		586.3
				3		515.3

St – standard, IS – internal standard
Bold letter with* denotes labelled arginine (R) or lysine (K) with a stable isotope ^{13}C and ^{15}N

3. *Cellular uptake of compounds **1-15** into immortalized microglia (BV2 cells)*

<u>Compound 1</u>

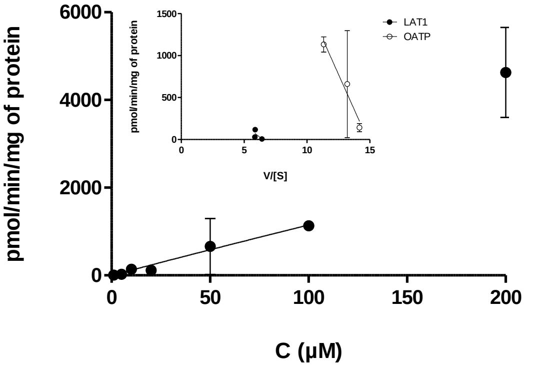

<u>Compound 2</u>

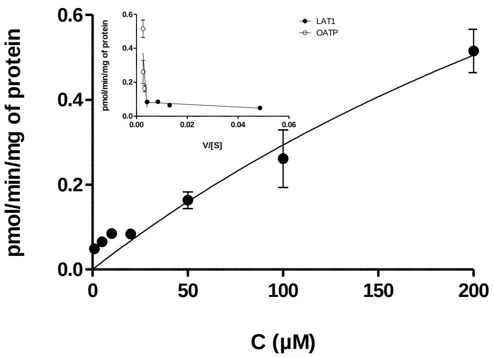

<u>Compound 3</u>

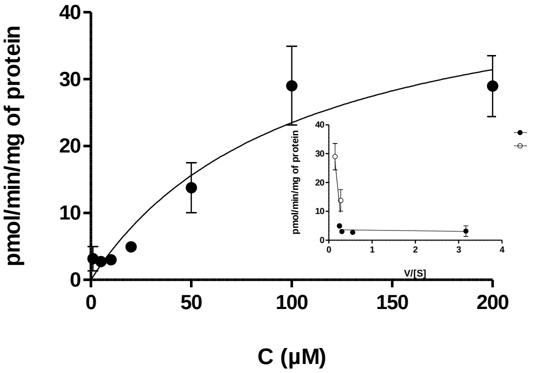

<u>Compound 4</u>

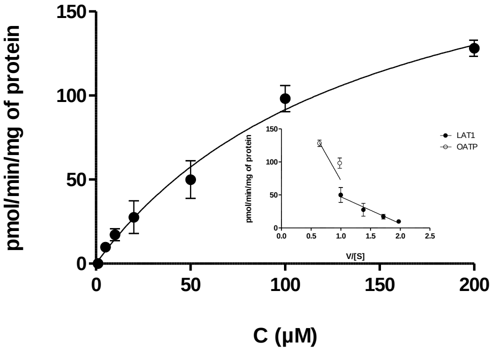

Compound 5

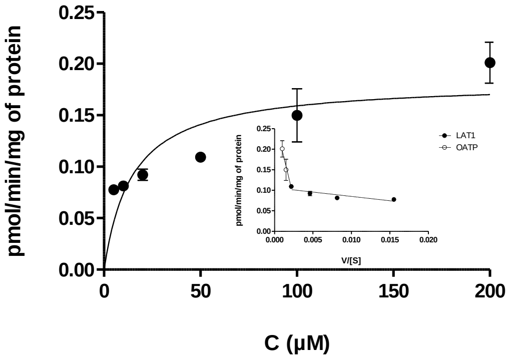

Compound 6

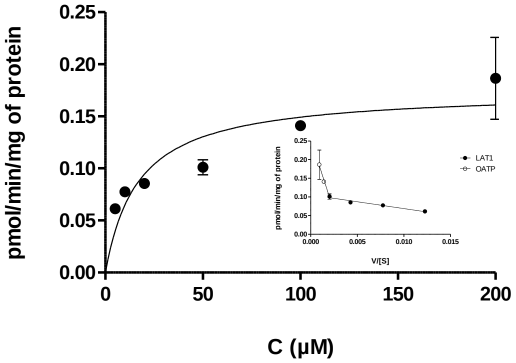

Compound 7

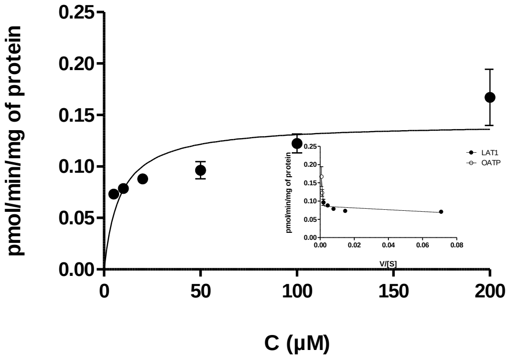

Compound 8

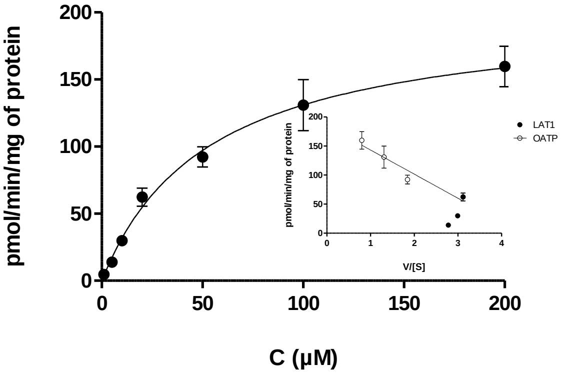

Compound 9

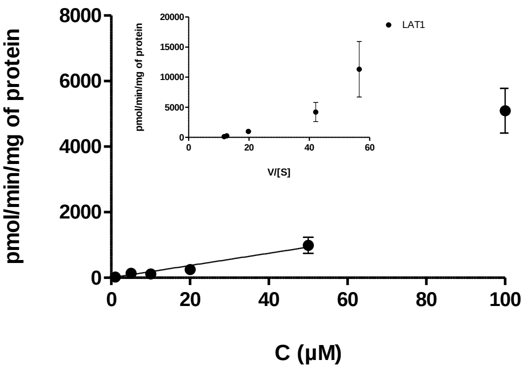

Compound 10

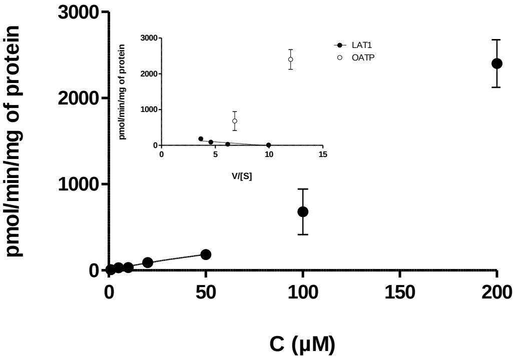

Compound 11

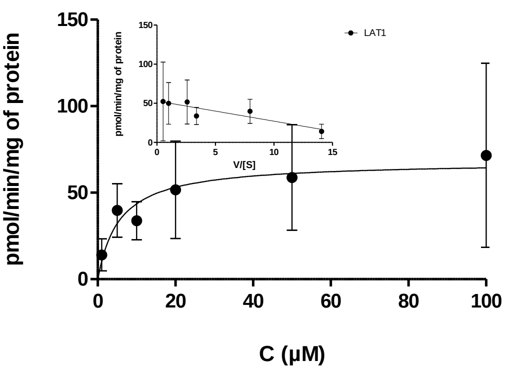

Compound 12

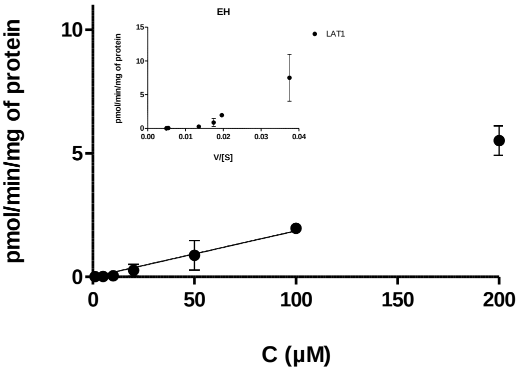

Compound 13

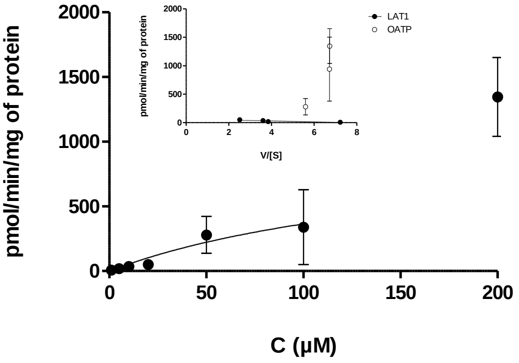

Compound 14

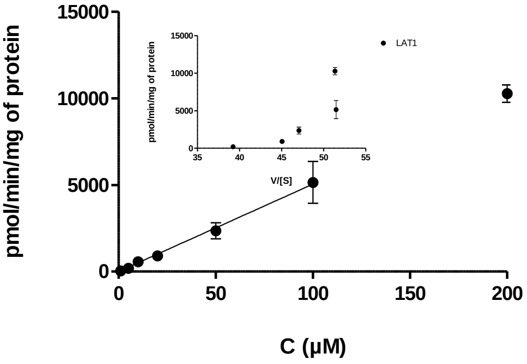

Compound 15

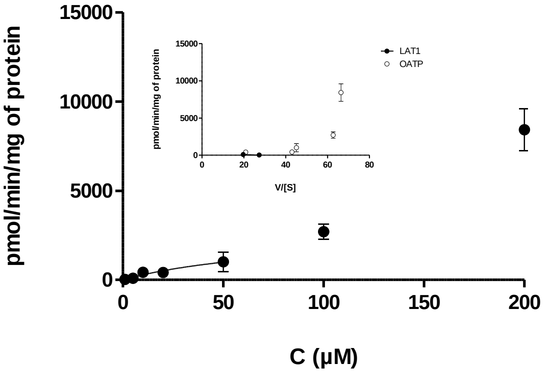

Figure S1. Cellular uptake of 1-200 μM compounds **1-15** into immortalized microglia (BV2 cells) after 30 min incubation (mean ± SD, n=3).

*4. Cellular uptake of compounds **1-15** in the absence and presence of LAT1-inhibitor*

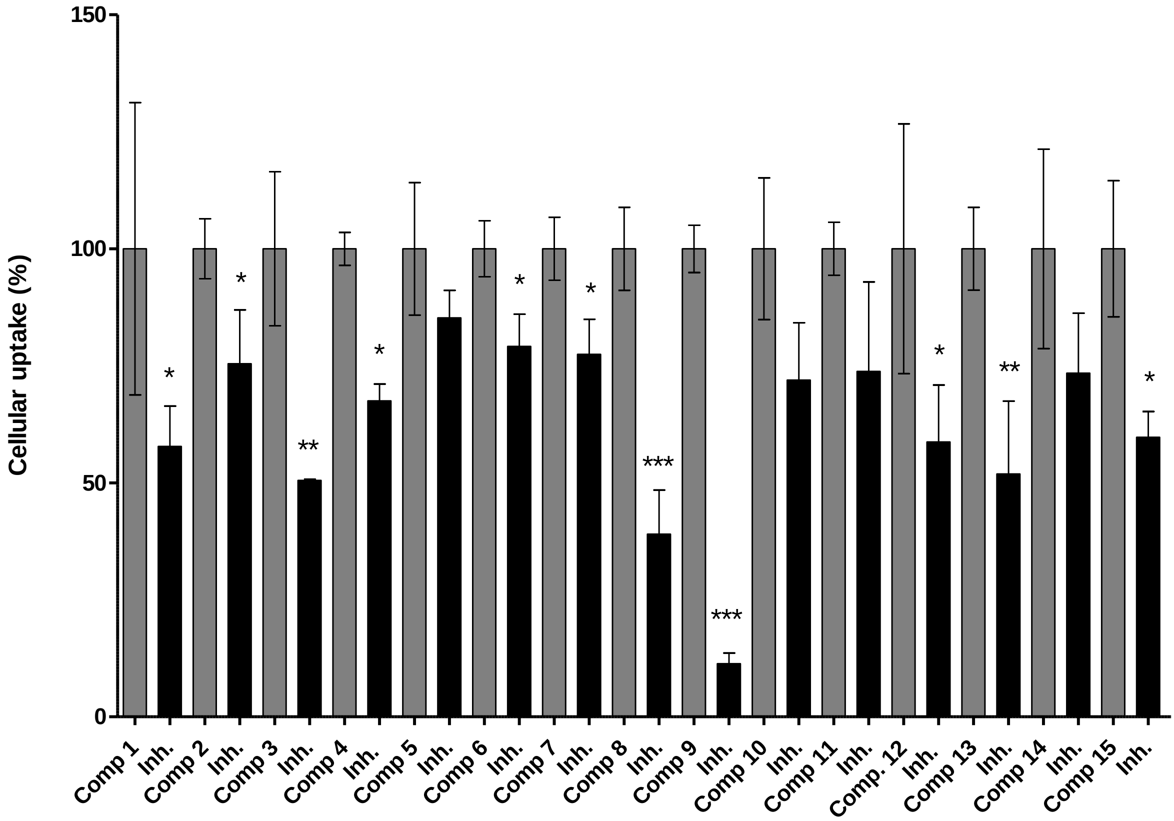

Figure S2. Cellular uptake of 100 µM compounds **1-15** into immortalized microglia (BV2 cells) with and without LAT1-inhibitor (KMH-233) after 30 min incubation (mean ± SD, n=3).

5. *Cellular uptake of [^{14}C]-L-leucine after incubation of compounds **1-15***

Compound 1

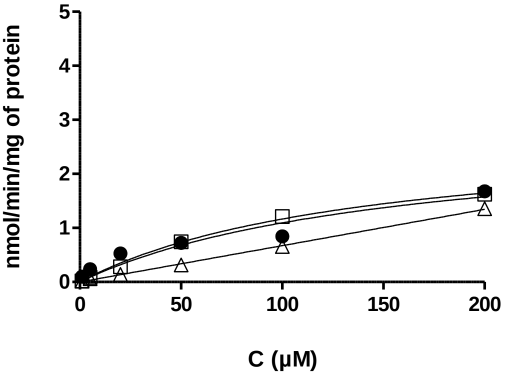

Compound 2

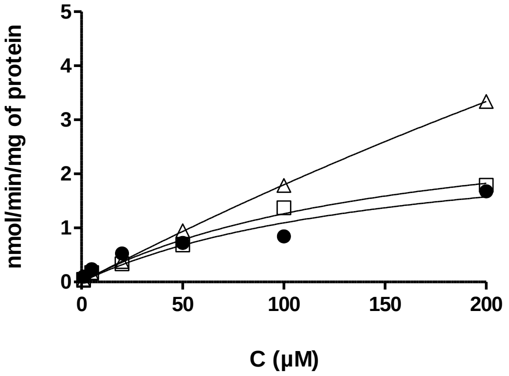

Compound 3

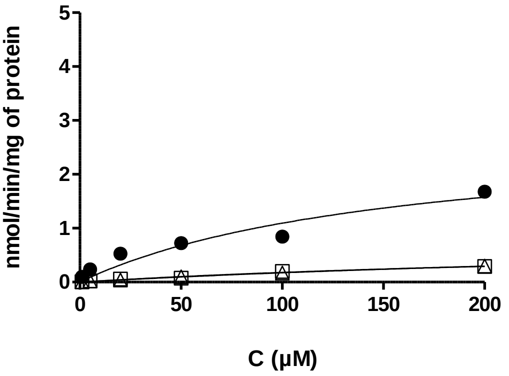

Compound 4

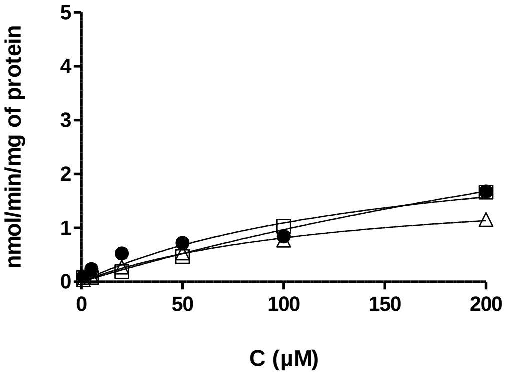

Compound 5

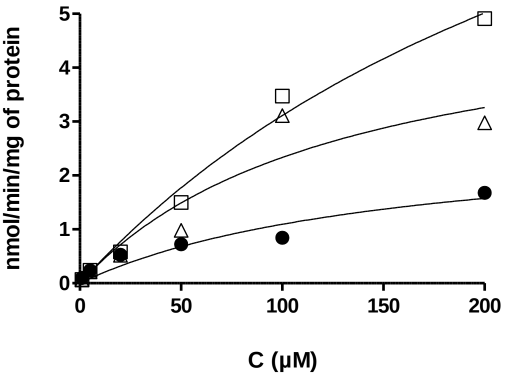

Compound 6

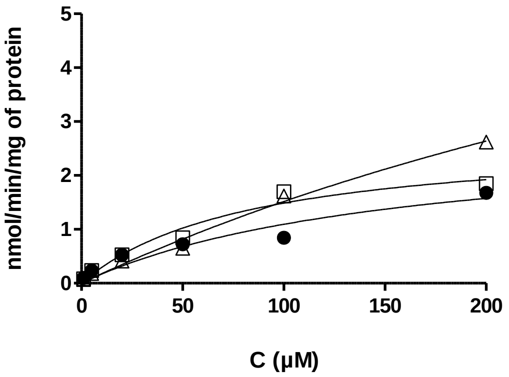

Compound 7

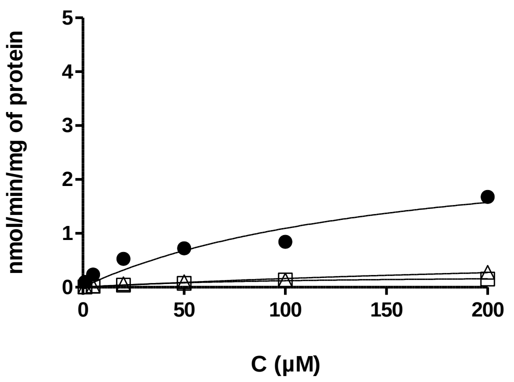

Compound 8

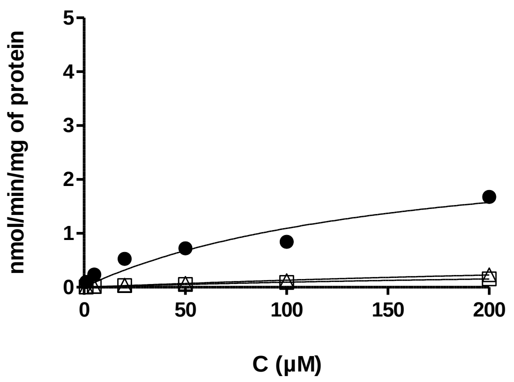

Compound 9

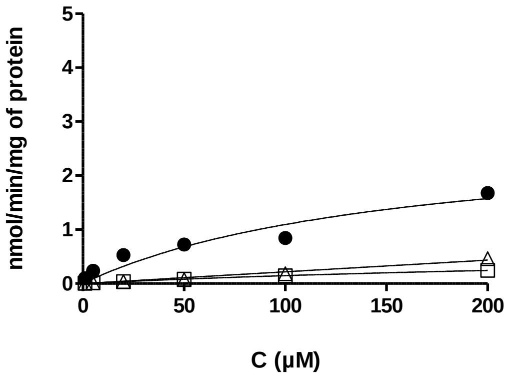

Compound 10

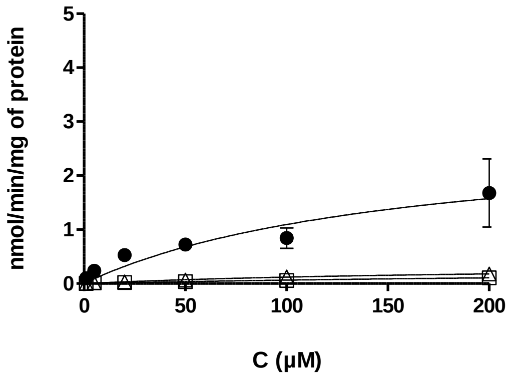

Compound 11

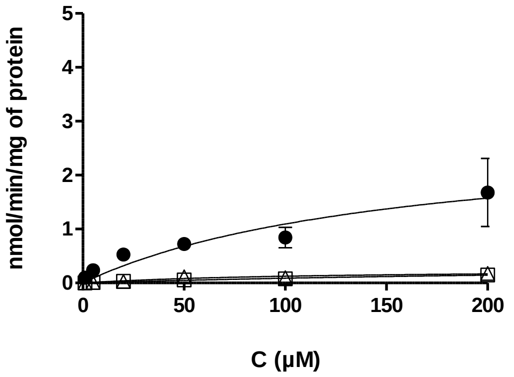

Compound 12

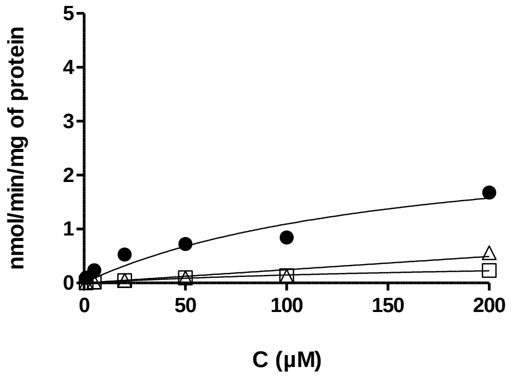

Compound 13

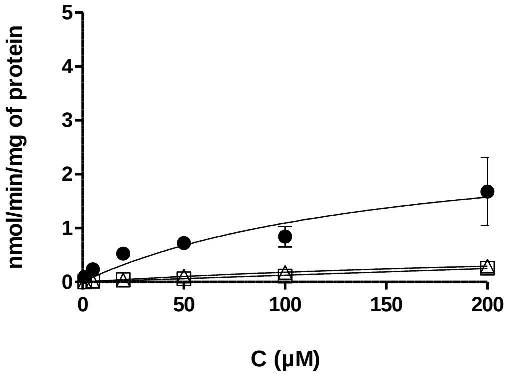

Compound 14

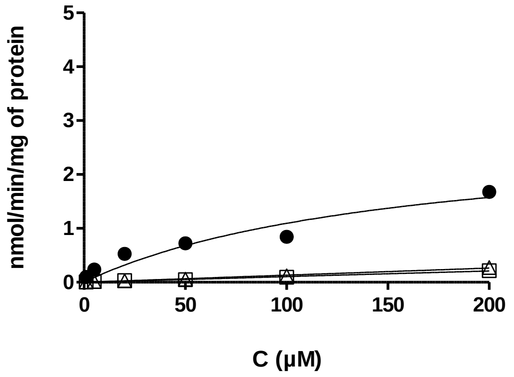

Compound 15

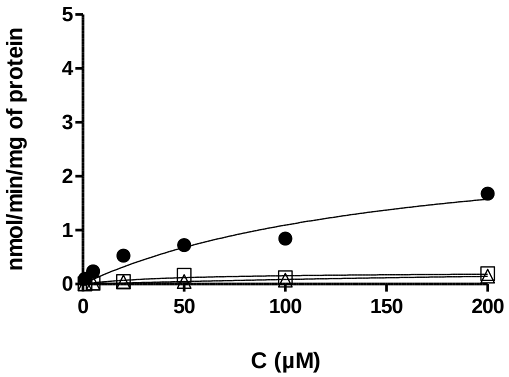

Thyroxin

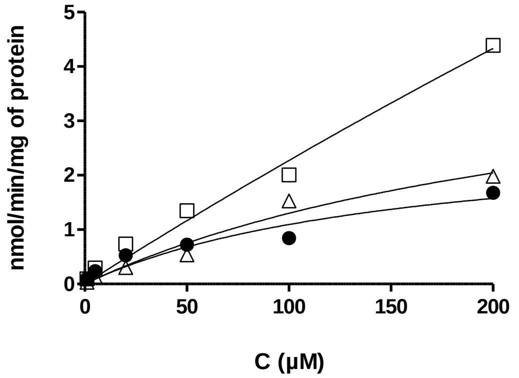

Dexamethasone

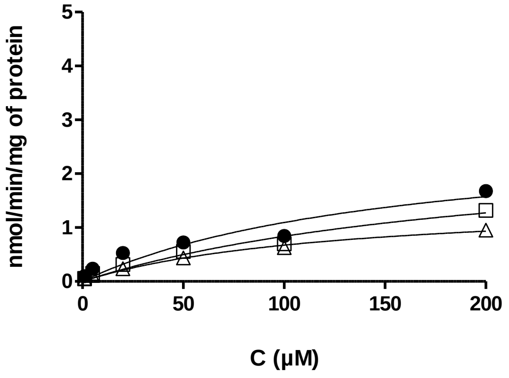

Phenobarbital

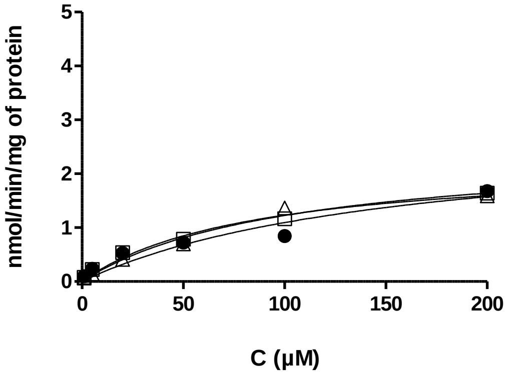

L-Glutamine

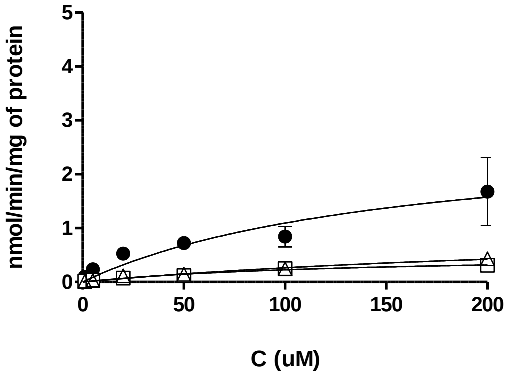

Figure S3. Cellular uptake of 1-400 µM [^{14}C]-L-leucine (● filled circles) into immortalized microglia (BV2 cells) after 10 min incubation (□ open squares) or 3 h incubation (Δ open triangles) of compounds **1-15**, thyroxin, dexamethasone, phenobarbital, or L-glutamine (mean ± SD, n=3).
